# Superfast Vocal Muscles Control Song Production in Songbirds

**DOI:** 10.1371/journal.pone.0002581

**Published:** 2008-07-09

**Authors:** Coen P. H. Elemans, Andrew F. Mead, Lawrence C. Rome, Franz Goller

**Affiliations:** 1 Department of Biology, University of Utah, Salt Lake City, Utah, United States of America; 2 Department of Biology, University of Pennsylvania, Philadelphia, Pennsylvania, United States of America; 3 The Marine Biological Laboratory, Woods Hole, Massachusetts, United States of America; Università di Parma, Italy

## Abstract

Birdsong is a widely used model for vocal learning and human speech, which exhibits high temporal and acoustic diversity. Rapid acoustic modulations are thought to arise from the vocal organ, the syrinx, by passive interactions between the two independent sound generators or intrinsic nonlinear dynamics of sound generating structures. Additionally, direct neuromuscular control could produce such rapid and precisely timed acoustic features if syringeal muscles exhibit rare superfast muscle contractile kinetics. However, no direct evidence exists that avian vocal muscles can produce modulations at such high rates. Here, we show that 1) syringeal muscles are active in phase with sound modulations during song over 200 Hz, 2) direct stimulation of the muscles *in situ* produces sound modulations at the frequency observed during singing, and that 3) syringeal muscles produce mechanical work at the required frequencies and up to 250 Hz *in vitro*. The twitch kinematics of these so-called superfast muscles are the fastest measured in any vertebrate muscle. Superfast vocal muscles enable birds to directly control the generation of many observed rapid acoustic changes and to actuate the millisecond precision of neural activity into precise temporal vocal control. Furthermore, birds now join the list of vertebrate classes in which superfast muscle kinetics evolved independently for acoustic communication.

## Introduction

Some of the most complex vocal communication signals in the animal kingdom are produced by songbirds [1], whose songs often contain long sequences of rapidly modulated sound elements [2]. Rapid acoustic modulations (<10 ms) during song can arise from passive interactions between the two independent (i.e. left and right) sound generators in the vocal organ – the syrinx [3] and intrinsic nonlinear dynamics of sound generating structures [e.g. 4]–[7]. However, many acoustic features of song correlate with neural [8]–[14] and electromyographic (EMG) activity [15]–[19], which suggests the possibility of direct neuromuscular control of the syrinx.

Indirect evidence from EMG recordings in brown thrashers (*Toxostoma rufum*) indicates that sound modulations up to 125 Hz correlate with muscle activity [17]. Furthermore, the variation in temporal characteristics of song in zebra finches (*Taenopygia guttata*) correlates with variation in the spiking patterns of neurons in premotor brain nuclei [8], [9], [11], [12], [14], which suggests that the temporal precision of the CNS can be expressed at the behavioral level of song production. Both findings are consistent with very fast muscular control of the vocal production system of songbirds. However, to actuate these rapid changes, songbirds would need to have evolved syringeal muscles with superfast contractile kinetics. This rarely evolved trait would enable them to produce positive work over 100 Hz [20]. To our knowledge no direct evidence exists that avian vocal muscles can produce modulations at such high rates.

To assess how high temporal precision in the central premotor song circuits is translated into equally high precision at the behavioral level, we need to make a direct determination of temporal performance limits of vocal muscular control in songbirds. By conducting a series of experiments at different levels of organization, we show that the vocal muscles of songbirds exhibit superfast contractile kinetics and can generate acoustic modulations up to 250 Hz.

## Results

**Figure 1:**
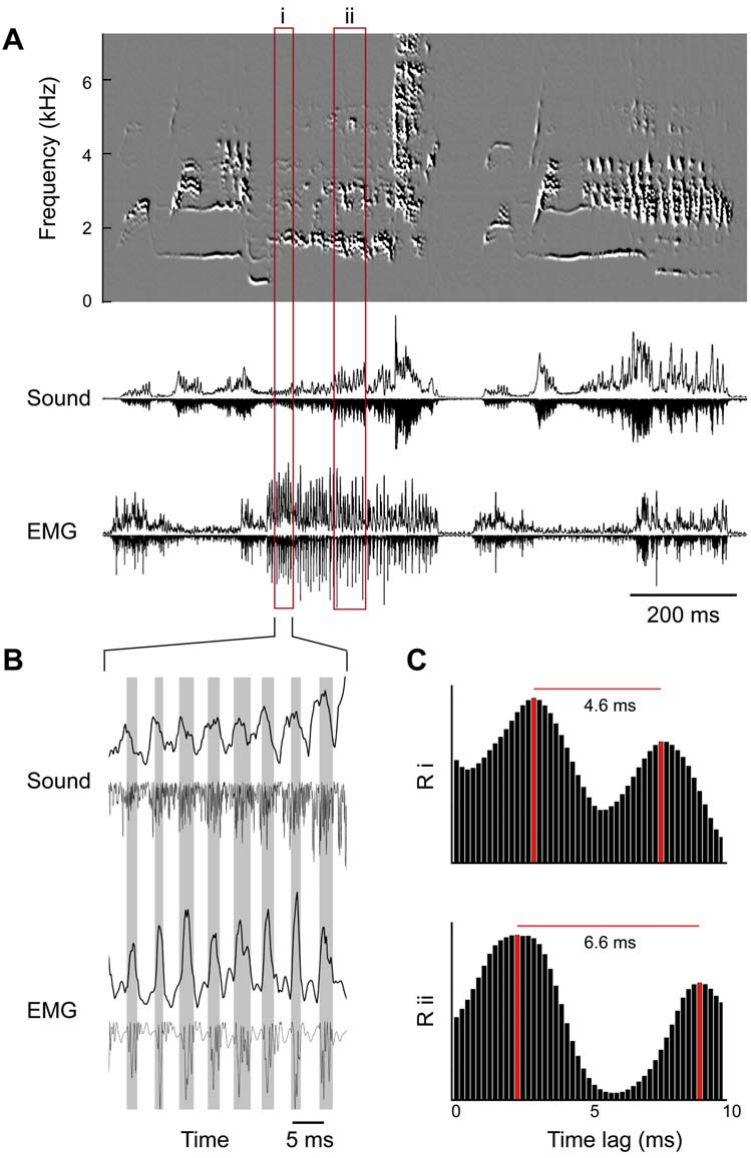
Song of starling exhibits fast modulations that correlate with muscle activity. (A) Spectral derivative plot [45] (top), oscillogram of sound and EMG activity of *m. tracheobronchialis dorsalis* (dTB). (B) Expanded time scale of segment i. The rate of modulation of the sound amplitude is paralleled by peaks in muscle activity (shaded areas). Positive traces; integrated values (time constant 0.2 ms), negative traces; half-wave rectified values. (C) Cross-correlations (R) of integrated sound amplitude and EMG activity signals of segments i and ii show a temporal link between EMG activity and sound modulation. The segments i and ii are indicated in (A). The distance between the local peaks in the bar diagrams equals the period of the signals. The periods are 4.6 and 6.6 ms in segment i and ii respectively, which is equivalent to repetition rates of 218 and 152 Hz. EMG; electromyogram of dTB, Sound; sound oscillogram.

European starlings (*Sturnus vulgaris*) have a complex learned song [21], [22], which contains many fast modulations. Some syllables contain amplitude modulated “buzzes” (100–125 Hz) and other modulations up to 200 Hz (Figure 1). In order to test whether these modulations could be the result of direct muscular control, we measured electromyographic (EMG) activity of the syringeal muscles in freely singing starlings (see Supporting Information, Methods S1). The main muscle causing amplitude modulations by regulating airflow is the *m. tracheobronchialis dorsalis* (dTB) [17]. *In vivo* recordings of dTB activity in freely singing starlings showed that amplitude modulation of the produced sound was accompanied by synchronized dTB activity, suggesting active control (Figure 1B). The dTB showed activity bursts that correlated with sound amplitude at intervals as short as 4.6 ms, which is equivalent to a repetition rate of 218 Hz (Figure 1C). If syringeal muscles cause these sound modulations, it requires that they can produce work and modulate syringeal parameters at these high rates.

To determine whether the muscles are indeed capable of this performance, we measured the *in vitro* performance of syringeal muscles in European starlings and zebra finches. We used isolated fibre bundles of the adductor muscle dTB in starlings and the abductor muscle *m. tracheobronchialis ventralis* (vTB) in zebra finches.

**Figure 2:**
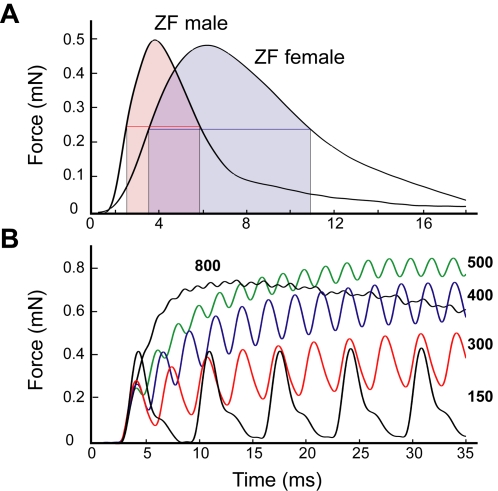
*In vitro* isometric properties of syringeal muscles exhibit superfast twitch kinetics. (A) Twitch characteristic of male and female zebra finch *m. tracheobronchialis ventralis* demonstrate extremely fast rise, and decay times. In zebra finches, syringeal muscle performance is sexually dimorphic. (Twitch half time 3.73±0.68 ms (male, N = 8) and 7.08±0.79 ms (female, N = 3). t-test; p<0.01) (B) Twitches of male starling *m. tracheobronchialis dorsalis* are completely separated at 150 Hz. Force summates at faster stimulation, and the tetanic fusion is not complete until 800 Hz. The stimulation frequencies corresponding to the curves are indicated in bold.

The twitch half-times measured 3.23±0.44 ms in male starling (N = 12), 2.93±0.79 ms in female starling (N = 3), 3.73±0.68 ms in male zebra finch (N = 8) and 7.08±0.79 ms for female zebra finch (N = 3) at 41.4°C. The twitch half-times were not significantly different between males and females in starlings (t-test: *p* = 0.5802, combined measurement: 3.23±0.44 ms, N = 15), but were significantly different between males and females in zebra finches (t-test; *p*<0.01; Figure 2A).

In some bird species, song control is lateralized [23]–[27] with a tendency to predominantly generate amplitude (and frequency) modulations on the right side in the brown thrasher [17]. Therefore we focused on obtaining muscle preparations from the right side of the syrinx (See Supporting Information, Methods S1). However, we also obtained a number of muscle preparations from the left side of the syrinx (three for male starling and two for male zebra finch). The twitch half-times did not differ significantly for starling (t-test, *p* = 0.499; lumped data males and females) or zebra finch (t-test, *p* = 0.969; males only).

In starlings, the dTB developed isometric force from 10% to 100% in 1.74±0.32 ms (N = 15). In one preparation, the twitch half-time was as short as 1.6 ms and it developed force from 10% to 100% in 1.03 ms. Twitches were still completely separate at 150 Hz stimulation frequency and tetanic fusion was not complete until 600–800 Hz in all preparations (Figure 2B).

**Figure 3:**
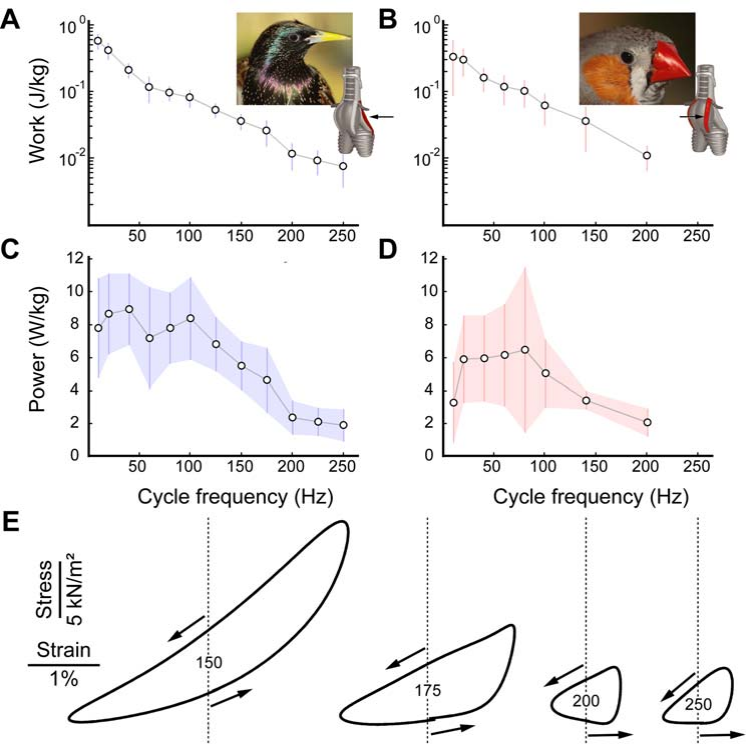
Superfast syringeal muscles produce positive work and power up to 250 Hz *in vitro*. (A) Work and (C) power production of male starling *m. tracheobronchialis dorsalis* (dTB, N = 5). (B) Work and ((D) power production of male zebra finch *m. tracheobronchialis ventralis* (vTB, N = 6). Plotted values are mean±S.D. The insets show the bird species and the position of the dTB and vTB muscle on a schematic of the songbird's syrinx (modified after [46]). The arrows indicate which muscle is being tested. (E) Active workloops of starling dTB at 150, 175, 200 and 250 Hz. Dotted vertical lines indicate zero strain ( = resting length). Arrows indicate lengthening (pointing right) and shortening (pointing left) portions of the workloop.

Isometric measurements, however, do not provide evidence whether the muscles can produce work at the high cycle frequencies as suggested by our *in vivo* measurements. To modulate sound, muscle must be able to perform mechanical work at the modulation frequency. Non-isometric measurements in which we subjected the muscles to various strain cycles and stimulation regimes (the workloop technique [28]) revealed that syringeal muscles indeed produce positive work and power at cycle frequencies up to 250 Hz (Figure 3). These data establish that syringeal muscles have the contractile potential to actuate syringeal elements as fast as 250 Hz.

**Figure 4:**
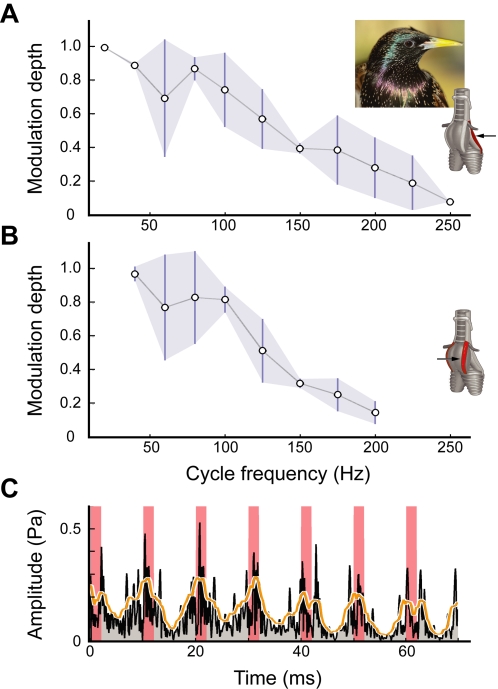
Stimulation of syringeal muscles causes tracheal flow and sound modulation. *In situ* muscle stimulation of syringeal muscles (A) dTB (N = 3) and (B) vTB (N = 3) modulates tracheal flow up to 250 Hz. Values are mean±S.D. The insets show the position of the dTB and vTB muscle on a schematic of the songbird's syrinx (modified after [46]). The arrows indicate which muscle is being tested. (C) Sound amplitude of vocalization during dTB stimulation at 100 Hz. The sound amplitude drops following muscle stimulation (red areas). Black trace; half-wave rectified sound signal, orange trace; integrated sound signal (time constant 2 ms).

Because measurements on isolated fiber bundles do not take into account additional elasticity and mass of the *in situ* configuration, we tested whether this extreme modulation performance is attainable by the whole muscle in the intact syrinx. We electrically stimulated the dTB and vTB at different rates and measured their capacity to modulate airflow in anaesthetized male starlings (Methods S1, Figure S1). These measurements showed that also *in situ*, syringeal gating muscles could modulate syringeal airflow up to 250 Hz (Figure 4A,B). When flow was increased above the phonation threshold, muscle stimulation caused amplitude modulation in the radiated sound (Figure 4C).

## Discussion

We present direct evidence that songbird have superfast syringeal muscles, which can modulate song acoustics up to 250 Hz. The extremely fast performance of isolated muscle fibers *in vitro* translates into very rapid modulation of syringeal airflow and sound amplitude in the biomechanically relevant setting of the intact syrinx *in situ*. Both tests indicate clearly that the presumed direct muscular control inferred from EMG activity during spontaneous song *in vivo* is well within the temporal performance capabilities of syringeal muscles, and therefore most likely indicates direct active neuromuscular control of sound modulation in spontaneously singing birds.

The performance of syringeal muscle ranks them among the fastest known vertebrate muscles [20]. With twitch contraction halftimes of 3.23±0.44 ms (N = 15) and 3.73±0.68 ms (N = 8) for the adductor muscle dTB in starling and the abductor muscle vTB in male zebrafinch, respectively, these highly specialized muscles attain the fastest measured isometric twitch kinematics of any vertebrate muscle to our knowledge. The isometric twitch characteristics of syringeal muscles in a non-songbird, the ring dove (*Streptopelia risoria*), were previously shown to be close to other superfast vertebrate muscles [15], but their performance limits (i.e., capability of performing mechanical work at high frequencies) were not explored [16]. With twitch half-times around 10 ms [15], the syringeal muscles in ring doves are much slower than the syringeal muscles of songbirds and can not be fast enough to control the modulation rates we observe in songbirds. Consistent with this, ring doves exhibit modulation rates of only 25 Hz during their vocalizations [16].

The extremely rapid activation and relaxation phases of syringeal muscle contraction require that multiple ultra-structural and molecular systems must be in place and work in concert [20]. It is currently unknown how the extremely rapid kinetics is achieved in syringeal muscles. Because both activation and relaxation are fast, we can expect that the processes of Ca^2+^ release and cross-bridge attachment during the activation cycle, and Ca^2+^ reuptake, Ca^2+^ unbinding from troponin and cross-bridge detachment during deactivation [20], [29], [30] are extremely fast. Syringeal muscles operate at a high temperature of 41°C. The expected increase in kinetics induced by these high temperatures may not require as extreme adaptations as found in ectothermic animals, such as toadfish, who call at temperatures from 15–25°C. Nonetheless, syringeal muscles are much faster than locomotory muscles and likely involve novel myosin isoforms.

Superfast muscle is a muscle type that has only sporadically evolved, but appears in some vertebrate classes. The best-documented cases are the swimbladder muscles in various fishes [29]–[33] and the tail shaker muscles in rattle snakes [29], [34]. Fast muscles have been observed in mammals: laryngeal muscle [35], and extraocular muscles e.g. [36], [37], but mechanical measurements are yet to place them in the same league as the superfast muscles described above. The presence of superfast muscles in the avian vocal organ adds another independent case where these highly specialized muscles seem to have evolved in a sound production system. The function of the vocal muscles in mammals and birds differs from that of the swimbladder and tailshaker muscles. In mammals and birds, sound is produced by airflow-induced vibration of vocal folds in the larynx or labia in the syrinx, respectively. Vocal muscles of the mammalian larynx and avian syrinx do not generate sound pulses with each contraction, but they adjust vocal parameters that cause modulation of the flow-induced oscillations of vocal folds and labia.

Cross-bridge kinetics [30] and space constraints at the muscle ultra-structural level dictate a trade-off between force production and maximal attainable frequency at which positive work can be produced [31], [38], [39]. This trade-off makes only certain biomechanical systems amenable to deriving benefits from superfast muscles. Motor systems under selection for speed therefore need to reduce actuator mass. The energy content of sound waves is low and the production and modulation of sound generally involves manipulation of low masses (e.g. swimbladder, rattle, syringeal and glottal structures), compared with much heavier skeletal elements (e.g. appendages). In contrast to muscles in locomotory systems [40], muscles in sound production systems appear to be optimized for speed and not power [31].

We show that motor performance of syringeal muscles in zebra finches is sexually dimorphic: the twitch characteristics of a main gating muscle vTB are significantly different between females and males, with the vTB of the females being almost two times slower. This sexually dimorphic performance could arise from any of the above-mentioned muscular (ultra-) structural or molecular systems that affect contraction speed. In starlings however, no sexual dimorphism is found in muscle performance. These results are paralleled by singing behavior of these two species: in zebra finches only the males sing [41], [42], whereas in starlings both males and females sing [21].

The neuromuscular control of song production in the syrinx is a well-known example of lateralized behavior [23]–[27], [43]. Despite the low number of preparations, our data suggest that there is no lateralization in syringeal muscle twitch performance in the two investigated species. Therefore both sides of the syrinx seem to have an equal potential to modulate labial vibrations.

Extremely rapid transitions during song - in the order of 1 ms - can be caused by intrinsic dynamics of the syrinx [4]. In this study we provide direct evidence that songbird syringeal muscles are sufficiently fast to actively modulate acoustic parameters at these same high rates. Syringeal muscles of starlings generate full force within 1.74±0.32 ms (N = 15), which allows direct adjustments of vocal output in the millisecond range. In addition, with the use of fast syringeal muscles timing of sound onset can be controlled with high precision. The vocal production system of birds operates with high fidelity and is capable of millisecond temporal precision in portions of the song [8], [10]–[12]. Superfast vocal muscles represent the mechanical actuator to translate the temporal precision in neural motor activity into similar precision in the behavioral output.

## Materials and Methods

### *In vivo* muscle activity

Syringeal muscle activity was measured in freely singing male starlings (*Sturnus vulgaris*) as previously described [16]–[19], [44]. All experiments were in accordance with the Institutional Animal Care and Use Committee (IACUC) of the University of Utah, Salt Lake City, USA. Methods are described in detail in the Online Supporting Information, Methods S1.

### *In vitro* muscle performance

The work and power generated by syringeal muscles was determined using the workloop technique on isolated muscle fibre bundles as previously described [16], [28], [29], [31]. In starlings, we isolated fibre bundles along the surface of *musculus tracheobronchialis dorsalis* (dTB). In zebra finches, we isolated fibre bundles of the *musculus tracheobronchialis ventralis* (vTB). Experiments were performed at the University of Pennsylvania, in October 2006 (zebra finches) and May 2007 (starlings) according to regulations by the IACUC, University of Pennsylvania, Philadelphia, USA.

### *In situ* muscle stimulation and flow modulation

The effect of direct muscle stimulation (dTB) on syringeal airflow was measured on anaesthetized starlings *in situ*. Airflow above the syrinx was monitored with a custom-built direction sensitive flow probe, consisting of plastic tubing with two heated microbead thermistors in the lumen of the tube. Experiments were performed at the University of Utah, June 2007. All experiments were in accordance with the IACUC of the University of Utah, Salt Lake City, USA. A detailed description of the Materials and Methods can be found in the Supporting Information. Methods S1.

## Supporting Information

Methods S1 Supplemental Information: Materials and Methods.
(0.06 MB DOC)

Figure S1 Flow modulation in the trachea following electrical stimulation of the syringeal muscles.
(1.10 MB TIF)
