# Supplementary material for: Superfast Vocal Muscles Control Song Production in Songbirds
Source: PLoS One. 2008 Jul 9;3(7):e2581. doi: 10.1371/journal.pone.0002581 (PMC2440420; doi:10.1371/journal.pone.0002581)
Supplement: Methods S1 — Supplemental Information: Materials and Methods. (0.06 MB DOC) [file pone.0002581.s001.doc]

Superfast Vocal Muscles Control Song Production in Songbirds

Coen P.H. Elemans, Andrew F. Mead, Lawrence C. Rome, Franz Goller

Supporting Information

Materials & Methods

**Animals.**

Seventeen male and eight female European starlings (*Sturnus vulgaris*) were caught in Salt Lake City, USA in January 2007. All animals were housed individually on a seasonally varied light:dark cycle and fed *ad libitum*. For the *in vitro* muscle experiments, ten male and three female zebra finches (*Taenopygia guttata*) were used. All zebra finches were housed in 45x45x52 cm group cages on a 12h:12h light:dark cycle and fed *ad libitum*.

***In vivo* muscle activity**

Syringeal muscle activity was measured in freely singing male starlings. The techniques for measuring muscle activity in freely singing birds have been described in detail [1-5] and will only be summarized here. All experiments were in accordance with the Institutional Animal Care and Use Committee (IACUC) of the University of Utah, Salt Lake City, USA. Male starlings were anaesthetized with an intramuscular injection of chloropent (3.4 μl/g). The syrinx was exposed through an incision in the skin and the interclavicular air sac between the clavicles. Up to four pairs of insulated bipolar EMG electrodes (0.025 mm stainless steel, California Fine Wire, CA) were inserted into syringeal muscles and secured to the outermost fascia with cyanoacrylate tissue adhesive (Nexaband, Abbott laboratories, IL, USA). Wires were led out of the air sac and routed subcutaneously to the back. The air sac was then closed with surgical sutures and Nexaband tissue adhesive. Spontaneous vocalizations were recorded with a microphone in front of the cage. EMG signals from the syringeal muscles were filtered and differentially amplified (Brownlee Precision, Instrumentation Amplifier 410 or 440, CA, USA). All signals were digitized at 32 kHz and recorded using AviSoft Recorder (Avisoft Bioacoustics, Berlin, Germany).

*Data analysis*

Spectral time derivatives were estimated using the methods described in ref. [6]. We used the Matlab code written Sigal Saar and Parta Mitra (available at *http://ofer.sci.ccny.cuny.edu/html/sam.html*). Sound amplitude and EMG signals were integrated with a time constant of 0.2 ms. We calculated the un-scaled cross-correlation estimates of the latter two signals:$R_{xy}(m)=\sum_{n=0}^{N-m-1} x_{n+m}y_{n}^{*}$ (for *m* ≥ 0), where *x* is the integrated sound amplitude signal, *y*^*^ is the complex conjugate of the integrated EMG signal, *N* is the length of the vectors and *m* is an integer variable (*m* = 1, …, 2*N*-1) to delay the signals with respect to each other (i.e. the lag variable).

***In vitro* muscle performance**

## Muscle preparations

The work and power generated by syringeal muscles was determined using the workloop technique [7]. Here, we provide a brief description of this technique, our protocol and calculations. Experiments were performed at the University of Pennsylvania, in October 2006 (zebra finches) and May 2007 (starlings) according to regulations by the IACUC, University of Pennsylvania, Philadelphia, USA. Animals were anaesthetized and euthanised using a 70/30% CO_2_/O_2_ mixture. The syrinx was exposed by cutting through the rib cage, isolated and pinned down on Sylgard-covered Petri dishes in oxygenated Ringers solution (recipe *cf*. [1,8,9]) at room temperature (21°C). In starlings, we isolated fiber bundles along the surface of *musculus tracheobronchialis dorsalis* (dTB). In zebra finches, we isolated fiber bundles of the *musculus tracheobronchialis ventralis* (vTB). In some bird species, song control is lateralized [10-14] and there is a tendency to predominantly generate amplitude (and frequency) modulations on the right side in the brown thrasher (*Toxostoma rufum*) [2]. Therefore we focused on obtaining muscle preparations from the right side of the syrinx. During the experimental protocol, we always kept the left side ready for dissection and oxygenized in case we had time left after the first preparation or something went wrong with the first preparation during the protocol. As such, in starlings (males and females), we obtained twitch data from 12 right and 3 left preparations (all from males). In zebrafinch males, we obtained twitch data from 6 right and 2 left preparations.

*Set-up and data acquisition*

Muscle preparations were mounted in a teflon-coated aluminum test chamber [15,16] that was continuously flushed with oxygenated Ringers solution at 21°C using a peristaltic pump (Rabbit-Plus, Rainin Instrument Co., Woburn, MA). One end of the muscle was mounted to the arm of a servo-motor (Model 6023AFM, Cambridge Technology, Cambridge, MA) using single strands of a 0G silk suture (Ethicon, Somerville, NJ) under a Wild M3 dissection microscope. The other end was mounted on a force transducer (Model 400, Cambridge Technology, Cambridge, MA). The position of the servo and force transducer could be controlled using micromanipulators. Preparations were stimulated with parallel platinum electrodes. Stimuli were applied with a pulse generator (Puslar 6b, FHC, Brunswick, ME) coupled to a DC power amplifier (Hewlett Packard, Palo Alto, CA). The temperature of the aluminum block with the test chamber was controlled using water-cooled Peltier elements (bipolar controller, Cambion, Cambridge, MA).

Force transducer, servo motor position and stimulation signals were digitized at 40 kHz with AD board PCI-MIO-16E4 (National Instruments, USA). We developed custom software in Matlab 7.3 (The Mathworks) to control the set-up and for data analysis.

## Experimental protocol

After a preparation was allowed to rest for 10 minutes, tetanic contractions (stimulation frequency 250 Hz, duration 100 ms, pulse duration 1.0 ms) were performed at 2-minute intervals to optimize stimulation parameters and resting length (*L*_0_) for maximal force generation. Then the temperature was slowly increased to 40.4 °C over a period of 15 minutes. After another rest period (5-10 minutes), twitch and tetanic (stimulation frequency 600-800 Hz, duration 50 ms, pulse duration 0.1 ms) characteristics were determined.

Muscle preparations were subjected to a series of 5-10 sinusoidal strain cycles around *L*_0_. The first and last cycles were omitted from analysis to avoid on- and offset transients. A run consisted of a series with stimulation (i.e. active series), followed by a series without stimulation (i.e. passive series). The performance of every preparation was optimized for power production at cycle frequencies of 10, 20, 40, 60, 80, 100, 125, 150, 175, 200, 225, 250 and 275 Hz by altering strain amplitude, stimulation phase and duty factor. After the optimized settings were obtained for every cycle frequency, we collected the final dataset by repeating the measurements for every cycle frequency with the optimized settings. Reference twitches and tetani were obtained every 30 minutes to monitor decay of the preparation. The tetanic tension of a preparation after the final dataset was compared to the first measurement prior to optimization. No preparation decreased more in tension than 34% and the average decrease was 22 ± 10 % (mean ± S.D.) after 142 ± 16 minutes.

After testing, non-contractile and dead material was removed under a dissecting microscope. The remaining muscle mass was blotted on Kimwipes to remove any fluid and weighed (model C-35 microbalance, ATI Cahn Instruments, Boston, MA; precision ± 0.1 μg) to determine wet weight. Preparations were desiccated overnight in a small vial with Drierite (Hammond Drierite Company, OH), to determine dry weight.

## Data Analysis

We used similar parameters as previous authors [1,8,9,17,18] to characterize the twitch. Maximal isometric stress (MIS) was calculated as *F*_max_ /*A*_csa_ of the muscle, where the cross-sectional area *A*_csa_ was estimated from the resting length *L*_0_ and wet weight of the muscle fibers (assuming a constant density of 1060 kg/m^3^ from [19]). The instantaneous power was $P_{inst}=F\cdot\frac{dL}{dt}\text{ [W]}$. Work per cycle was defined as the area of the work loop: $W=\oint F\cdot dL$ [J]. Mean power was calculated as the product of mean net work and cycle frequency: $\overline{P}=\overline{W}\cdot f$ [W].

At cycle frequencies ≥100 Hz, a special precaution was taken to calculate work done by the muscle preparation. The strain wave that is forced on the muscle preparation by the servo motor, travels from the servomotor end to the force transducer end of the muscle preparation at a speed roughly depending on the Young’s modulus and density of the muscle. Therefore, at very high cycle frequencies (≥100 Hz), the force measured lags slightly behind the applied strain at the servomotor. This phase delay creates the false appearance of additional positive work done by the muscle. Because this phase-lag is present in both active and passive runs, we obtained work done by the active muscle by subtracting the aligned force signals of the passive set from those of the active set in workloops at cycle frequencies ≥100 Hz. The active muscle is stiffer than the passive muscle, which leads to a smaller lag in the active trials compared to the passive trials. Therefore the passive trial generates a larger false amount of work than the active trial. This method of subtraction results in an underestimation of work done by the active muscle at cycle frequencies ≥100 Hz. Note that at cycle frequencies <100 Hz, this phase lag is insignificant (passive work was negative or zero) and no subtraction was made.

*In situ* flow modulation

## Surgical procedures

Experiments were performed at the University of Utah, June 2007. All experiments were in accordance with the Institutional Animal Care and Use Committee (IACUC) of the University of Utah, Salt Lake City, USA. Three male starlings were anaesthetized with an intramuscular injection of chloropent (3.4 μl/g). A 10 cm silastic tube cannula (OD, 2.16 mm; ID, 1.02 mm; cat. no. 508-005, Dow Corning, MI, USA) was inserted in the anterior thoracic air sac to control flow through the syrinx. The syrinx was exposed by making a 20 mm incision in the skin between the clavicles and opening the interclavicular air sac. Bipolar, teflon-coated silver-wire stimulation electrodes (76 μm diameter without coating, A-M systems Inc., WA, USA) were implanted in the rostral part of the left *musculus tracheobronchialis dorsalis* (dTB) and left *musculus tracheobronchialis ventralis* (vTB). The electrodes were glued to the muscle fascia with Nexaband tissue adhesive and routed out of the interclavicular air sac. The air sac was then closed with surgical sutures and tissue adhesive.

Airflow above the syrinx was monitored with a custom-built direction sensitive flow probe, consisting of plastic tubing with two heated microbead thermistors in the lumen of the tube. The trachea was exposed by making an incision in the neck rostral of the right clavicle. The flow probe was inserted in the trachea about 20 mm above the syrinx by removing a small piece of two tracheal rings. The trachea was closed with tissue adhesive and the skin closed with surgical sutures.

*Set-up and data acquisition*

After surgery, the bird was placed on its back in a pre-shaped bed of modeling clay in the bottom-centre of a 80x60x60 cm box, lined with 3 cm thick sound insulation foam (SONEX, Illbruck Acoustic, MN, USA). The beak was held open at constant angle with a 3 mm diameter tube. Sound was recorded 2-4 cm from the beak opening with a microphone (type 40AG, pre-amplifier type 26AK, power module 12AD, G.R.A.S., Denmark). Moisturized air was supplied through the air sac cannula with a digital mass-flow/pressure controller (Alicat Scientific, Tucson, AZ, USA). In addition, supplied air pressure was measured prior to the cannula connection, 10 cm from the air sac, with a 3.5F Mikro-Tip catheter pressure transducer (SPR524, Millar Instruments, Texas, USA) connected to a pre-amplifier and pressure control unit (PCU-2000, Millar Instruments, Texas, USA). The flow passing each thermistor was measured by a Wheatstone bridge circuit (Hector Engineering, IN, USA), where the current needed to maintain the heated thermistor at a constant temperature is proportional to the rate of airflow. The muscle stimulation electrodes were connected to two optically isolated, constant current stimulators (DS3, Digitimer Ltd. Hertfordshire, UK). Sound pressure, air sac pressure, the two flow signals and the mass flow and pressure from the flow controller were low-pass filtered at 10 kHz (Brownlee Precision, Instrumentation Amplifier 410, CA, USA) and digitized at 30 kHz with an AD board (PCI-6251, National Instruments, USA). The set-up was controlled with custom written software in Labview (National Instruments).

*Experimental protocol*

Above a certain bronchial pressure and or flow threshold, the labia in the syrinx start self-sustained oscillations [4]. We determined the phonation onset pressure *p*_on_ of every bird by stepwise increasing pressure *p* in a 6 s block wave (from atmospheric pressure to *p*, and back to atmospheric pressure). The fundamental frequency of the self-sustained oscillations of the labia was 250-500 Hz. When the muscle modulation is in the order of magnitude of the fundamental frequency of sound, distinguishing muscle modulation from labial oscillation becomes impossible using the radiated sound signal and difficult using the tracheal flow signal. Figure 4**c** show an example of sound modulation in starling B15, stimulating the dTB at 100 Hz. Therefore, we used a pressure below onset phonation threshold (0.90 x *p*_on_) to study flow modulation by the syringeal muscles.

Both dTB and vTB were stimulated using periodic pulse train stimulations (5 -20 cycles) at cycle frequencies of 20, 40, 60, 80, 100, 125, 150, 175, 200, 225 and 250 Hz, with a tetanic frequency of 600 Hz. Pulse duration was optimized for the 20 Hz cycle frequency and kept constant at 0.5 –1.0 ms for the rest of the protocol. Measurements for every cycle frequency were optimized for modulation depth of the tracheal flow by optimizing the number of pulses in the pulse train (i.e. duty factor of the cycle) and pulse amplitude (0.5 – 2.0 mA). After the experiment, the cannula, stimulation electrodes and flow probe were removed. The two flow signals from the probe were calibrated by applying known flow amplitudes using the mass-flow/pressure controller (Alicat Scientific, AZ, USA).

## Data Analysis

Muscle stimulation results in flow modulation (Figure S1). Stimulating vTB increases flow, while stimulating the dTB reduces flow. Modulation depth per stimulus (*i*) is defined as: $D_{i}=\frac{min\text{(F}_{\text{i}}\text{) - }max\text{(F}_{\text{i}}\text{) }}{\text{F}_{\text{pre }}\text{- }min\text{(F}_{\text{i}}\text{) }}$, where *min*(F_i_) and *max*(F_i_) are the local minima and maxima in the flow signal (F) associated with stimulus *i*, and *F*_pre_ is the average flow value of the 50 ms segment prior to stimulation. In figure S1, *min*(F_i_) are indicated by blue diamonds, *max*(F_i_) by green diamonds and F_pre_ by the red boxes. The average modulation depth is calculated as: $\overline{D}=\frac{\sum_{3}^{n-2} D_{i}}{n}$, where *n* is the number of stimuli.

FIGURE LEGEND

**Figure S1.** Flow modulation in the trachea following electrical stimulation of the syringeal muscles. Upper trace, *musculus tracheobronchialis dorsalis* (dTB); lower trace, *musculus tracheobronchialis ventralis* (vTB). Blue diamonds indicate local minima, green diamonds indicate local maxima in the flow signal associated with preceding stimulus. Red boxes indicate flow value prior to stimulation (*F*_pre_) for dTB and vTB. Orange boxes on top and bottom of figure indicate muscle stimuli for dTB and vTB respectively.

REFERENCES

1. Elemans CPH, Spierts ILY, Hendriks M, Schipper H, Müller UK, van Leeuwen JL (2006) Syringeal muscles fit the trill in ring doves (*Streptopelia risoria* L.). *J Exp Biol* 209: 965-977.
2. Goller F, Suthers RA (1996) Role of syringeal muscles in gating airflow and sound production in singing brown thrashers. *J Neurophysiol* 75: 867-876.
3. Goller F, Suthers RA (1996) Role of syringeal muscles in controlling the phonology of bird song. *J Neurophysiol* 76: 287-300.
4. Suthers RA, Goller F, Hartley RS (1994) Motor dynamics of song production by mimic thrushes. *J Neurobiol* 25: 917-936.
5. Vicario DS (1991) Contributions of syringeal muscles to respiration and vocalization in the zebra finch. *J Neurobiol* 22: 63-73.
6. Tchernichovski O, Nottebohm F, Ho CE, Pesaran B, Mitra PP (2000) A procedure for automated measurement of song similarity. *Anim Beh* 59: 1167 - 1176.
7. Josephson RK (1985) The mechanical power output from striated muscle during cyclic contraction. *J Exp Biol* 114: 493-512.
8. Elemans CPH, Spierts ILY, Muller UK, van Leeuwen JL, Goller F (2004) Superfast muscles control dove’s trill. *Nature* 431: 146.
9. Askew G, Marsh RL (2001) The mechanical power output of the pectoralis muscle of blue-breasted quail (*Coturnix chinensis*): the *in vivo* length cycle and its implications for muscle performance. *J Exp Biol* 204: 3587-3600.
10. Goller F, Suthers RA (1995). Implications for lateralisation of birdsound from unilateral gating of ipsilateral motor patterns. *Nature* 373: 63-66.
11. Nottebohm F (1971) Neural lateralization of vocal control in a passerine bird. I. Song. *J. Exp. Zool*. 177: 229-262.
12. Nottebohm F (1971) Neural lateralization of vocal control in a passerine bird. II. Subsong, calls and a theory of vocal learning. *J. Exp. Zool*. 179: 35-50.
13. Suthers RA (1990). Contributions to birdsong from the left and right sides of the intact syrinx. *Nature* 347: 473-477.
14. Suthers RA (1998) Peripheral control and lateralization of birdsong. *J. Neurobiol* 33: 632-652.
15. Rome LC, Swank D (1992) The influences of temperature on power output of scup red muscle during cyclical length changes. *J Exp Biol* 171: 261-281.
16. Rome LC, Swank D, Corda D (1993) How fish power swimming. *Science* 261: 340.
17. Rome LC, Syme DA, Hollingworth S, Lindstedt SL, Baylor SM (1996) The whistle and the rattle: The design of sound producing muscles. *Proc Natl Acad Sci USA* 93: 8095-8100.
18. Young IS, Rome LC (2001) Mutually exclusive designs: the power output of the locomotory and sonic muscles of the oyster toadfish (*Opsanus tau*). *Proc R Soc Lond B* 268: 1965-1070.
19. Méndez J, Keys A (1960) Density and composition of mammalian muscle. *Metabolism* 9: 184-188.
