# Supplementary figures and images for: Superfast Vocal Muscles Control Song Production in Songbirds
Source: PLoS One. 2008 Jul 9;3(7):e2581. doi: 10.1371/journal.pone.0002581 (PMC2440420; doi:10.1371/journal.pone.0002581)

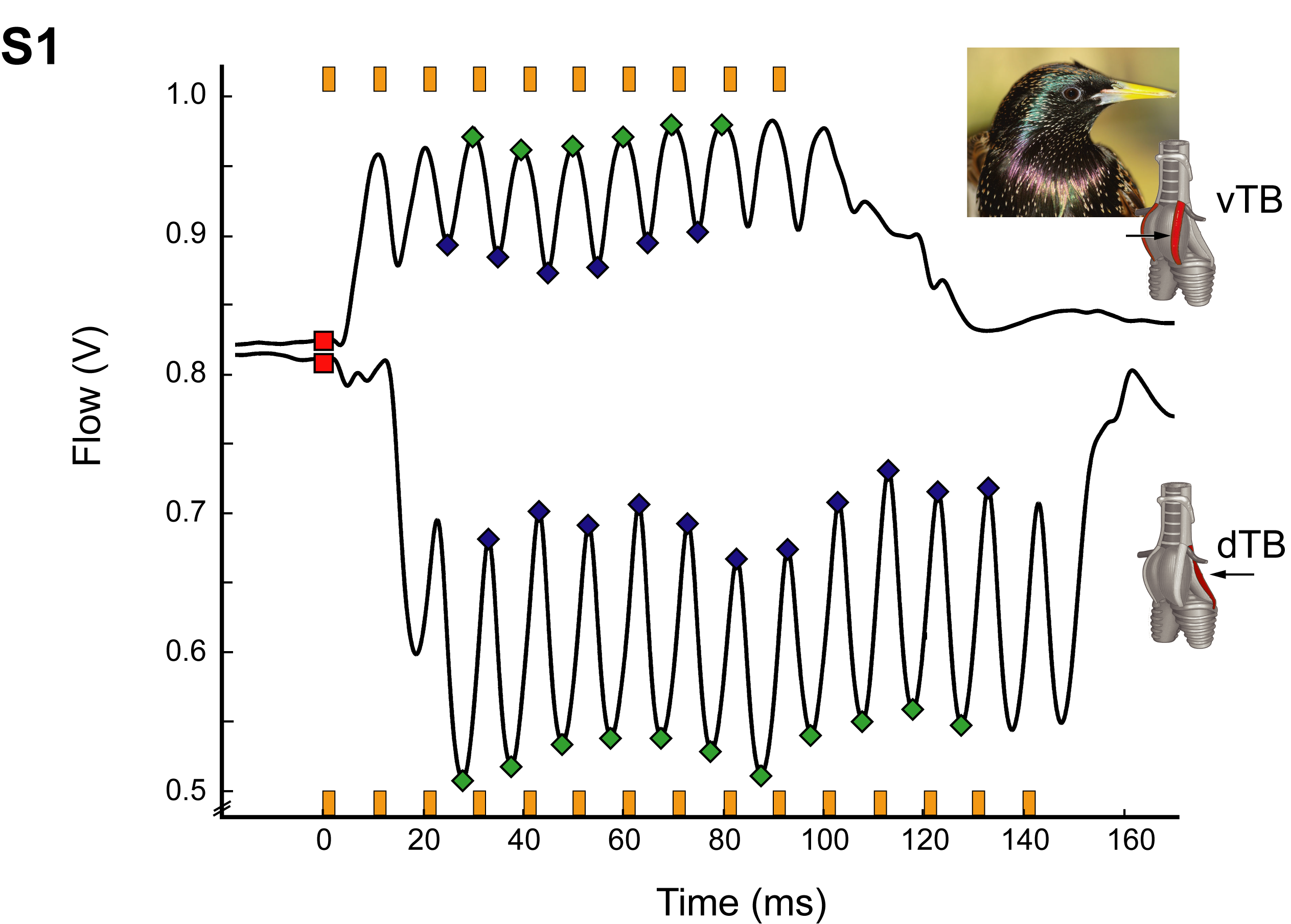

Supplement: Figure S1 — Flow modulation in the trachea following electrical stimulation of the syringeal muscles. (1.10 MB TIF) [file pone.0002581.s002.tif]
